# Change in composition of the *Anopheles gambiae* complex and its possible implications for the transmission of malaria and lymphatic filariasis in north-eastern Tanzania

**DOI:** 10.1186/1475-2875-11-188

**Published:** 2012-06-08

**Authors:** Yahya A Derua, Michael Alifrangis, Kenneth M Hosea, Dan W Meyrowitsch, Stephen M Magesa, Erling M Pedersen, Paul E Simonsen

**Affiliations:** 1 National Institute for Medical Research, Amani Centre, P. O. Box 81, Muheza, Tanzania; 2 Department of International Health, Centre for Medical Parasitology, Immunology and Microbiology, University of Copenhagen, Copenhagen, Denmark; 3 Department of Molecular Biology and Biotechnology, University of Dar es Salaam, Dar es Salaam, Tanzania; 4 Department of Public Health, University of Copenhagen, Copenhagen, Denmark; 5 DBL- Centre for Health Research and Development, University of Copenhagen, Copenhagen, Denmark; 6 RTI International, Centre for Strategic Malaria Solutions, Global Health Group, Nairobi, Kenya

**Keywords:** *Anopheles gambiae* s.s., *An. arabiensis*, Longitudinal survey, Malaria, Lymphatic filariasis, Tanzania

## Abstract

**Background:**

A dramatic decline in the incidence of malaria due to *Plasmodium falciparum* infection in coastal East Africa has recently been reported to be paralleled (or even preceded) by an equally dramatic decline in malaria vector density, despite absence of organized vector control. As part of investigations into possible causes for the change in vector population density, the present study analysed the *Anopheles gambiae* s.l. sibling species composition in north-eastern Tanzania.

**Methods:**

The study was in two parts. The first compared current species complex composition in freshly caught *An. gambiae* s.l*.* complex from three villages to the composition reported from previous studies carried out 2–4 decades ago in the same villages. The second took advantage of a sample of archived dried *An. gambiae* s.l*.* complex specimens collected regularly from a fourth study village since 2005. Both fresh and archived dried specimens were identified to sibling species of the *An. gambiae* s.l. complex by PCR. The same specimens were moreover examined for *Plasmodium falciparum* and *Wuchereria bancrofti* infection by PCR.

**Results:**

As in earlier studies, *An. gambiae* s.s*.*, *Anopheles merus* and *Anopheles arabiensis* were identified as sibling species found in the area. However, both study parts indicated a marked change in sibling species composition over time. From being by far the most abundant in the past *An. gambiae* s.s*.* was now the most rare, whereas *An. arabiensis* had changed from being the most rare to the most common. *P. falciparum* infection was rarely detected in the examined specimens (and only in *An. arabiensis*) whereas *W. bancrofti* infection was prevalent and detected in all three sibling species.

**Conclusion:**

The study indicates that a major shift in *An. gambiae* s.l*.* sibling species composition has taken place in the study area in recent years. Combined with the earlier reported decline in overall malaria vector density, the study suggests that this decline has been most marked for *An. gambiae* s.s*.*, and least for *An. arabiensis*, leading to current predominance of the latter. Due to differences in biology and vectorial capacity of the *An. gambiae* s.l. complex the change in sibling species composition will have important implications for the epidemiology and control of malaria and lymphatic filariasis in the study area.

## Background

Malaria is the most prevalent mosquito borne disease posing a potential health risk to almost half of the world’s population. The World Health Organization estimated that approximately 216 million people were infected worldwide in 2010 resulting in an estimated 655,000 deaths, of which 91% were in the African region [1]. In the past few years, reports have indicated a decrease in malaria cases and related deaths in several endemic countries in Africa [1-3]. Although this decline has been attributed to the scale-up of effective malaria control interventions [1], some reports indicate that in some areas it preceded it [4]. Of particular interest in this respect is the decline in malaria in north-eastern Tanzania, which has been reported to occur in parallel with a dramatic decline in anopheline mosquitoes in an area with no organized vector control [5,6]. The prevalence of lymphatic filariasis, another infection transmitted by anopheline mosquitoes, is also currently decreasing in north-eastern Tanzania. Much of this decrease is due to an ongoing mass drug administration programme [7], but the decrease in anopheline mosquitoes may also be a contributing factor.

The *Anopheles gambiae* s.l. complex serves as an important vector of both malaria and lymphatic filariasis on the East African coast. The complex comprises of seven well recognized sibling species which have only slight morphological differences (not sufficient to make clear distinction between them) but they vary in their ecology and behaviour which is directly reflected in their vectorial capacity [8-11]. Studies conducted on the East African coast have identified *Anopheles gambiae* s.s., *Anopheles arabiensis* and *Anopheles merus* to be the local sibling species in the *An. gambiae* s.l. complex [12-17]. The two fresh water breeding species (*An. gambiae* s.s. and *An. arabiensis*) differ in their degree of host preference, with the first being strongly anthropophilic [18-20], while the later is more liberal and exhibit zoophilic tendencies especially when alternative mammalian hosts are available [9,19]. In areas where domestic animals are kept outside, *An. arabiensis* has been reported to have a greater tendency of feeding and resting outdoors, and it has been shown in such areas that human blood index and sporozoite rates are much lower in *An. arabiensis* as compared to *An. gambiae* s.s. [21]. Other studies have moreover indicated that *An. gambiae* s.s. lives longer than *An. arabiensis*[22,23]. The salt-water breeding species, *An. merus* has been considered a non-vector with exophilic and zoophilic tendencies [19], but more recent studies have shown that it can be a vector for both malaria and lymphatic filariasis [12,24]. Specifically for the coast of north-eastern Tanzania, past research has documented the predominance of *An. gambiae* s.s. over the two other members of the complex [12,14,25-27].

Due to their diverse ecology and behaviour, identification of individual sibling species of the *An. gambiae* s.l. complex is of paramount importance for understanding the epidemiology of the infections they transmit and for setting up the most appropriate control interventions. The observed overall decline in the anopheline population [6] may have affected the sibling species of the *An. gambiae* s.l. complex differently, resulting in change in the sibling species composition and thereby in the transmission characteristics. This study was designed to analyze for a possible change in the relative abundance of members of the *An. gambiae* s.l. complex over time (in light of the decline in overall anopheline population) as basis for better understanding the decline in malaria and lymphatic filariasis transmission in north-eastern Tanzania.

## Methods

### Study area

Mosquitoes were collected in four coastal villages in Tanga Region of north-eastern Tanzania: Kwale (S 04.96981°; E039.13639°), Vyeru (S 04. 95754°; E 039.13483°) and Tawalani (S 04.87785°; E 039.14295°) located approximately 20, 22 and 36 kilometers north of Tanga City along the Tanga-Mombasa road, and Kirare (S 05.24943°; E 039.02876°) located approximately 20 kilometers south of Tanga City along the Tanga-Pangani road. The villages are all located along the coast of the Indian Ocean (within 2 kilometers from the sea) and have fairly similar topography and weather conditions. The inhabitants practice subsistence farming and fishing and keep domestic animals like cattle, goats and chicken. Most of the houses are mud-walled with thatched roof, but few brick-walled and iron-roofed houses are also seen in Kwale, Vyeru and Kirare. No major mosquito control measures have been implemented in recent years in any of these villages, but insecticide impregnated bed-nets were distributed to every household shortly after completion of the present study (in September 2011).

### Mosquito collection and study design

The *An. gambiae* s.l. complex composition in Kwale, Vyeru and Tawalani has been analysed on several occasions in the past [12,14,25-27]. These analyses showed that *An. gambiae* s.s., *An. merus* and *An. arabiensis* are the sibling species present in the area, with the first of these by far the most abundant and the last by far the least abundant. A study was therefore set up to analyse the current complex composition in these villages, and to compare this to the composition reported in the past (Study A). For this purpose, mosquitoes were collected from 10 houses in each of the three villages during the peak mosquito production season in early June 2011. In order to optimize mosquito collection, a purposive sampling approach was used. In all parts of the village, households with thatched roof and open eaves, and located close to mosquito breeding sites, were selected. Mosquitoes were collected every night for two weeks (total of 14 nights) using Centre for Disease Control (CDC) light traps (John W. Hock Company, P. O. Box 12822, Gainesville, FL 32604, USA) hung beside an occupied, untreated bed-net. The traps were switched on at 1900 hours and off at 0600 hours by trained field assistants. Caught mosquitoes were transferred to paper cups and transported to the laboratory for identification using morphological criteria. Mosquitoes identified to belong to the *An. gambiae* s.l. complex were stored individually in perforated plastic vials and kept in sealable plastic bags containing silica gel desiccant.

Kirare has been the focus of an intensive study on lymphatic filariasis transmission and control [6,7]. Since the start of the study in 2003, mosquitoes have been collected once weekly from 50 originally randomly selected households using CDC light traps. The method for collection has been described in detail elsewhere [7], but essentially has been similar to that described above for Kwale, Vyeru and Tawalani. When back in the laboratory, mosquitoes were identified and all live filarial vector mosquitoes were dissected for *W. bancrofti* filaria infection. Since January 2005, all the dead filarial vector mosquitoes were kept dried and archived in Eppendorf tubes containing silica gel desiccant. These dried and archived mosquitoes provided a unique opportunity to examine the change in composition of the *An. gambiae* s.l. complex over time. A study was, therefore, set up to analyse the complex composition of the 281 available archived dried *An. gambiae* s.l. specimens (Study B). For the purpose of examining the change in composition over time these mosquitoes were divided into two groups, namely those collected between January 2005 and December 2006 (Study B1) and those collected between January 2007 and April 2011 (Study B2).

After sibling species determination, the specimens from both Study A and B were moreover examined for presence of *Plasmodium falciparum* and *Wuchereria bancrofti* infections.

### Deoxyribonucleic acid (DNA) extraction from mosquitoes

DNA extractions were carried out by using either the Qiagen DNeasy Kit (Qiagen Inc. Mississauga, Canada) or the Bender buffer method with modifications as described earlier [28]. The two methods were both found to work well in the subsequent PCR tests. In brief, Qiagen DNeasy Kit extraction involved homogenizing individual mosquitoes in 200 μl of Phosphate-Buffered Saline (PBS). 200 μl of buffer AL and 20 μl of proteinase K were added and the solution was incubated at 70°C for 10 minutes. An additional 20 μl of proteinase K was then added, followed by incubation at 56°C for one hour. This was followed by centrifugation at maximum speed (14,000 rpm) for 5 minutes. The supernatant was transferred to new Eppendorf tubes and mixed with 200 μl of 98% ethanol, and the entire sample was loaded into a DNeasy mini-column. This was followed by centrifugation at 11,000 rpm for 1 minute during which DNA bound to the DNeasy column membrane and other materials passed through. The DNA was washed three times to remove any contaminants and thereafter eluted with elution buffer. Bender buffer extraction involved homogenizing individual mosquitoes in 100 μl of Bender buffer (0.1 M NaCl, 0.2 M Sucrose, 0.1 M Tris–HCl pH 7.5, 0.05 M EDTA pH 9.1, 0.5% SDS) pre-heated at 65°C. After incubation at 65°C for 30 minutes, 15 μl of pre-chilled 8 M potassium acetate was added and followed by additional incubation on ice (at 4°C) for another 30 minutes. The lysate was then centrifuged at 14,000 rpm for 5 minutes and the supernatant was transferred to new tubes. This was followed by addition of 250 μl of pre-chilled absolute ethanol, incubation at −21°C for 3 hours and centrifugation at 10,000 rpm for 10 minutes. The supernatant was removed, and the pellet was washed with 70% ethanol, air dried and re-dissolved in sterile double distilled water and stored at −20°C until use.

### PCR for identification of *An. gambiae s.l.* sibling species, *P. falciparum* infection and *W. bancrofti* infection

A previously developed method [29], based on species-specific nucleotide sequences found in the ribosomal DNA intergenic spacers was used to identify members of the *An. gambiae* s.l. complex. The method uses five oligonucleotide primers to identify *An. gambiae* s.s.*, An. arabiensis, Anopheles quadriannulatus, Anopheles melas* and *An. merus,* and runs as a multiplex PCR*.* PCR reactions were conducted in a final volume of 20 μl consisting of 0.25 μM of each of the five primers, 1:1 TEMPase Hot Start polymerase master mix (Ampliqon III, VWR-Bie Berntsen, Denmark, including MgCL_2_ containing buffer and dNTP, according to manufacturer's instructions) and 2 μl of DNA extract. The samples were amplified in a VWR™ DuoCycler (VWR International bvba, Geldenaasksebaan 464, B-3001 Leuven) and cycling conditions were 95°C for 15 minutes followed by 35 cycles of denaturation at 94°C for 30 seconds, annealing at 50°C for 30 seconds, extension at 72°C for 30 seconds and final extension at 72°C for three minutes.

To identify *P. falciparum* infection in the mosquitoes, the DNA extracted from the individual specimens of *An. gambiae* s.l*.* was analysed by nested PCR as described [30] with the limitation that only the *P. falciparum* specific primers were used in the nested PCR assay. The outer PCR reactions were run in a total volume of 20 μl containing 0.0625 μM of each of the two primers (PLU 5&6), 1:1 Hot-Start TEMPase polymerase master mix and 2 μl of DNA extract. The outer DNA amplifications were run by VWR™ DuoCycler and the cycling conditions were 94°C for 15 minutes followed by 30 cycles of denaturation at 94°C for 1 minute, annealing at 58°C for 2 minutes, extension at 72°C for 2 minutes followed by final annealing and extension at 58°C for 2 minutes and 72°C for ten minutes, respectively. The resultant PCR products were used as a template in the nested PCR with *P. falciparum* primers, where each 20 μl of PCR reaction contained 0.25 μM of each of the two primers (rFAL 1&2), 1:1 Hot-Start TEMPase polymerase master mix and 1 μl of DNA extract. The cycling conditions were as described for outer PCR.

To identify *W. bancrofti* infection in the mosquitoes, the samples were examined by PCR for a highly repeated DNA sequences (the SspI repeat) found in *W. bancrofti* infections as previously described [31]. Each of the 20 μl of PCR reactions consisted of 0.25 μM of each of the two primers (NV1&NV2), 1:1 Hot-Start TEMPase polymerase master mix and 2 μl of DNA extract. The cycling conditions were 95°C for 15 minutes followed by 54°C for 5 minutes: then 35 cycles of denaturation at 94°C for 20 seconds, annealing at 54°C for 30 seconds, extension at 72°C for 30 seconds and final extension at 72°C for 5 minutes.

Each batch of samples in PCR were run with positive and negative controls and all PCR reactions were conducted in 96-well PCR plates covered with plate mats. The PCR products were mixed with 8 μl of loading dye (250 mg bromophenol blue, 40 g sucrose, made to 100 ml with distilled water) and 10 μl of the product loaded in 1.5% agarose gel premixed with ethidium bromide (2 mg/ml) stain. A marker of 50 bp ladder was also run on each gel for species identification and *P. falciparum* detection while a 100 bp ladder was used for *W. bancrofti*. Following gel electrophoresis, the PCR products were visualized using a BioRad gel image facility (Bio-Rad Laboratories) and the acquired gel image transferred to Quantity one program (Quantity one®, Bio-Rad Laboratories) for further analysis.

### Data analysis

All data on collected mosquitoes were entered in Excel and subsequently analyzed in IBM SPSS Statistics version 19.

## Results

### Freshly collected mosquitoes from Kwale, Vyeru and Tawalani (Study A)

**Figure 1:**
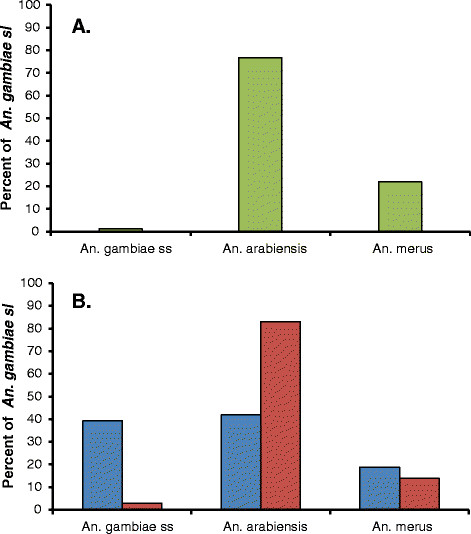
**Relative proportion of the three members of the *An. gambiae *s.l. complex.** (**A**): freshly collected specimens from Kwale, Vyeru and Tawalani in Study A; (**B**): archived specimens from Kirare in Study B (blue columns: Jan 2005 to Dec 2006, Study B1; red columns: Jan 2007 to Apr 2011, Study B2).

A total of 1,149 *An. gambiae* s.l. complex mosquitoes were collected from the three villages (43 from Kwale, 241 from Vyeru, 865 from Tawalani). Of these, 591 (all specimens from Kwale and Vyeru and an approximately similar number of randomly selected specimens from Tawalani) were processed for *An. gambiae* s.l. complex sibling species identification (Table1). Among the 585 identified specimens, by far the majority were *An. arabiensis* (76.8%), followed by *An. merus* (22.1%) and *An. gambiae* s.s*.* (1.2%) (Figure1A). A similar pattern of species composition was seen in each of the three individual villages, with a range of 70.6–83.7% for *An. arabiensis*, 15.5-27.7% for *An. merus* and 0.0-1.7% for *An. gambiae* s.s.

**Table 1:** **PCR testing of mosquitoes for *An. gambiae *complex sibling species, *P. falciparum *infection and *W. bancrofti *infection**

**Study**	**Collection period**	**No. mosquitoes tested**	**Type of PCR test**	**No. mosquitoes positive in PCR test**				
				***An. gambiae*s.s.**	***An. arabiensis***	***An. merus***	**Total identified**	**Not identified**
A	June 2011	591	*An. gambiae* s.l.	7	449	129	585	6
			*P. falciparum*	0	2	0	2	0
			*W. bancrofti*	0	10	3	13	0
B1	Jan 2005 – Dec 2006	166	*An. gambiae* s.l.	58	62	28	148	18
			*P. falciparum*	0	0	0	0	0
			*W. bancrofti*	5	3	3	11	3
B2	Jan 2007 – Apr	115	*An. gambiae* s.l.	3	83	14	100	15
	2011		*P. faciparum*	0	0	0	0	0
			*W. bancrofti*	0	6	0	6	1

Study A: freshly collected mosquitoes from Kwale, Vyeru and Tawalani; Study B: archived mosquitoes from Kirare.

An overview of the sibling species composition as seen in earlier studies from the three villages is shown in Table2. It is noted that in all these studies *An. gambiae* s.s. was found to be by far the most dominant species followed by *An. merus* and *An. arabiensis*. Thus a clear shift in sibling species composition appeared to have taken place between earlier studies carried out 26–38 years ago and the present study, with *An. gambiae* s.s*.* changing from being the most abundant to being the most rare, and *An. arabiensis* from being the most rare to being the most abundant.

**Table 2:** **Earlier studies on the *Anopheles gambiae *s.l. complex composition in Kwale, Vyeru and Tawalani**

**Study period**	**Study village**	**Catch method**	**Method for identification**	**No. mosquitoes in analysis**	***An. gambiae*sibling species identified and their proportion**	**Ref.**
1973 - 1975	Tawalani,	Pyrethrum spray catch &	Morphology, salinity test,	Not	An. gambiae s.s.*	[26]
	Kwale	human bait catch (indoor)	cytotaxonomy	reported	An. arabiensis*	
					An. merus*	
Aug 1975 – Dec 1977	Kwale	Human bait catch (indoor)	Cytotaxonomy	60	*An. gambiae s.s.*	[25]
					*+ An. merus* (98.3%)	
					*An. arabiensis* (1.7%)	
Jun – Dec 1977	Vyeru	Human bait catch &	Morphology, salinity test,	519	*An. gambiae s.s.*(53.0% )	[12]
		spray catch (indoor)	Cytotaxonomy		*An. merus* (40.5%)	
					*An. arabiensis* (6.6%)	
Apr 1982 – Sep 1983	Tawalani	Hand catch (indoor)	Cytotaxonomy	174	*An. gambiae* s.s. (92.5%)	[27]
					*An. merus*^¥^	
					*An. arabiensis* (7.5%)	
Apr 1982 – Jan 1985	Tawalani	Hand catch & pyrethrum	Cytotaxonomy,	196	*An. gambiae s.s.*(75.5%)	[14]
		spray catch (indoor)	electrophoresis		*An. merus* (17.4%)	
					*An. arabiensis* (7.1%)	

*No proportions given.

^¥^**Population of *An. merus*not analyzed by study villages.**

**Figure 2:**
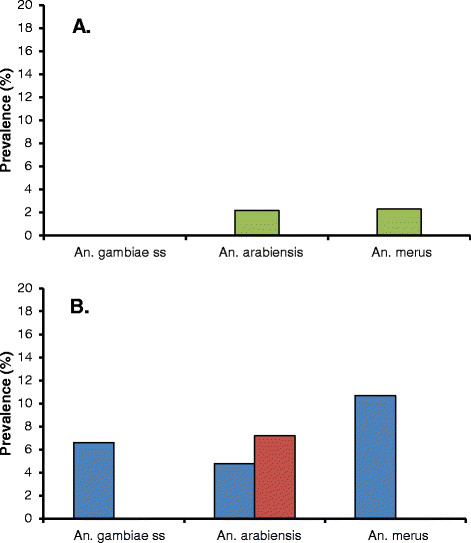
**Prevalence of *W. bancrofti *infection among members of the *An. gambiae *s.l. complex.** (**A**): freshly collected specimens from Kwale, Vyeru and Tawalani in Study A; (**B**): archived specimens from Kirare in Study B (blue columns: Jan 2005 to Dec 2006, Study B1; red columns: Jan 2007 to Apr 2011, Study B2).

Examination of the specimens for infection (Table1) showed that only two were positive for *P. falciparum* (both were *An. arabiensis*), whereas 13 were positive for *W. bancrofti*. The prevalence of *W. bancrofti* infection was approximately similar among the *An. arabiensis* (2.2%) and *An. merus* (2.3%) specimens, whereas no *An. gambiae* s.s. were found infected (Figure2A).

### Archived mosquitoes from Kirare (Study B)

The archived dried *An. gambiae* s.l. complex from Kirare village were divided into two groups (Table1), namely 166 specimens collected between January 2005 and December 2006 (Study B1) and 115 specimens collected more recently between January 2007 and April 2011 (Study B2). Among identified specimens, *An. gambiae* s.s*.*, *An. arabiensis* and *An merus* comprised 39.2%, 41.9% and 18.9%, respectively, during the earlier period, whereas during the later period the composition had changed markedly to 3.0%, 83.0% and 14.0%, respectively (Figure1B). Thus, again a clear shift in sibling species composition was observed, with *An. arabiensis* becoming relatively much more, and *An. gambiae* s.s. relatively much less, abundant when moving from the earlier to the later period. Statistical analysis indicated that the decrease in the proportion of *An. gambiae* s.s*.* and the increase in the proportion of *An. arabiensis* (in relation to the overall identified population of *An. gambiae* s.l. complex) were highly significant (Chi-square test, p < 0.001 for both tests).

Examination of the specimens for infection (Table1) showed no *P. falciparum* infection in any of the two groups, whereas 11 and 6 of the identified specimens were positive for *W. bancrofti* in the early (B1) and late (B2) collection group, respectively. The prevalence of *W. bancrofti* was rather similar in the three sibling species during the early collection period, whereas only *An. arabiensis* was positive for *W. bancrofti* during the late collection period (Figure2B).

## Discussion

This study documented the temporal change in relative abundance of sibling species of the *An. gambiae* s.l. complex in four villages in north-eastern Tanzania, by comparing the present composition to that seen in previous studies in the same villages and by analysis of archived specimens. Potential confounding associated with differences in sampling methods and in seasonal and spatial variation were taken into consideration when designing the study and analysing the findings. The mosquitoes were collected using CDC light traps, which are effective for indoor collection of host seeking mosquitoes, with a catch that compares well with the standard human landing catch method [32]. Considering the previously reported decline in anopheline mosquito density [6], it is noteworthy that the relative abundance of *An. gambiae* s.l. complex mosquitoes was considerably higher for the freshly collected mosquitoes in study A (average number per trap per night was 2.74) than what was earlier reported from Kirare [6] (annual averages of 0.2-0.3 *An. gambiae* s.l. per trap per night for 2006–2008). This might be due to the fact that mosquitoes for study A were collected during the peak mosquito production season while in Kirare mosquitoes were collected throughout the year. The fact that the abundance also varied considerably between the three villages in study A ( average of 6.2, 1.7 and 0.3 for Tawalani, Vyeru and Kwale villages, respectively) indicates that generalization of the entomological parameters across villages should be done with caution. As the employed light traps mainly collect indoor host-seeking mosquitoes, an attempt was made to assess the outdoor-biting activity by using two outdoor operated MosquitoMagnet™ traps (American Biophysics Corporation, East Greenwich RI) [33] in Vyeru and Tawalani during the same period. Only eight *An. gambiae* s.l. complex mosquitoes were caught in these traps during seven days and nights of trapping, indicating that outdoor biting by the members of the *An. gambiae* s.l. complex was at a rather low level in the study villages.

The analysis of the *An. gambiae* s.l. complex revealed, as in earlier studies, that *An. gambiae* s.s*.*, *An. merus* and *An. arabiensis* were the sibling species found in the study area. Of 585 specimens of the *An. gambiae* s.l. complex from study A identified to species level, over three quarters (76.8%) were *An. arabiensis*. When comparing the current composition with that reported 2–4 decades ago (Table2), there had been a marked change in sibling species composition over time. From being by far the most abundant in the past, *An. gambiae* s.s*.* was now the most rare, whereas *An. arabiensis* had changed from being the most rare to the most abundant. The findings on archived mosquitoes in study B confirmed that the process of change in composition of the *An. gambiae* s.l. complex also continued during more recent time. Thus, in the earlier survey period (study B1), *An. gambiae* s.s and *An. arabiensis* were in comparable proportions (39.2% vs 41.9%, respectively). In the later survey period (Study B2) an outstanding shift in composition was noted whereby the proportion of *An. gambiae* s.s. had decreased to only 7.7% of what it was in the earlier study period (Study B1) while that of *An. arabiensis* had almost doubled.

During the sampling period for study A (rainy season), the population of *An. gambiae* s.s. was expected to be at its peak, while that of *An. arabiensis* was expected to be low as it normally builds up gradually toward the dry period [9,34]. It has moreover earlier been reported [12] that *An. merus* and *An. gambiae* s.s. occured in approximately equal proportions during the rainy season where breeding sites for both species were plentiful, but as the fresh water pools dried up the population of the latter diminished while that of the earlier persisted. It therefore appears that the observed shift in sibling species composition of the *An. gambiae* s.l. complex was not simply due to seasonal variation, and the reason for the marked change in composition is unclear. Some studies have shown that malaria control interventions such as indoor residual spraying and use of insecticide treated nets while lowering the abundance of anopheline mosquitoes can sometimes also change the relative composition of the *An. gambiae* s.l. complex [35-38]. Although no organized vector control activities have been reported in the study areas, it cannot be excluded that even a limited distribution of insecticide treated nets to pregnant women may to some extent have affected the vector complex composition, as reported elsewhere [39,40]. However, it appears likely that additional more powerful environmental factors, such as climate change, pollution and/or change in socio-economic standards, may have played an important role.

Examination of the *An. gambiae* s.l. for *P. falciparum* revealed very few infections (overall rate of 0.24% in the 833 examined specimens), all of which were found in *An. arabiensis*. The low infection rate in vector mosquitoes agrees with the reported decline in parasitaemia in human beings [5]. In contrast, *W. bancrofti* infection was found in all the three sibling species, with an overall infection rate of 3.6% (Table1). It should be noted that these rates are based on all vector-borne stages of the parasite, since the PCR test used cannot distinguish between the different larval stages of *W. bancrofti*. Another cause for the high rate of *W. bancrofti* in the vectors could be a relatively high prevalence of microfilaraemia in the human population (reported to be 10.6% in Kirare village in October 2008 [7]) despite the ongoing programme for control of lymphatic filariasis. An earlier study in Tawalani village showed a malaria sporozoite rate of 0.37% and a *W. bancrofti* infection rate of 9.5% [27].

In the course of the PCR identification of members of the *An. gambiae* s.l. complex, 39 specimens (mainly from Kirare, see Table1) could not be identified using the current protocol. However, during detection of infection, 4 of unidentified specimens were positive for *W. bancrofti* indicating that probably we were dealing with a filarial vector mosquito that does not belong to the *An. gambiae* s.l. complex. Following subsequent inclusion of *Anopheles funestus* group primers, as described [41], 64.1% of the unidentified specimens (including the four *W. bancrofti* infected specimens) were found to belong to the *An. funestus* group (23 *An. funestus* s.s., one *Anopheles rivulorum* and one *Anopheles leesoni*). This emphasize the challenges involved in the identification of light trap collected anopheline specimens, as important morphological diagnostic features (maxillary palps and legs, important for separating *An. gambiae* s.l. complex and *An. funestus* group) are frequently damaged.

The change in composition of the *An. gambiae* s.l. complex obviously has important implications for the transmission of both malaria and lymphatic filariasis. The efficiency of transmission of these infections is closely related to the host choice, feeding habits and longevity of the vectors, which varies between the sibling species [20,23,24,42-44]. While transmission by the more anthropophilic, endophilic and long-living *An. gambiae* s.s. is declining, it is taken over by a more adaptable *An. arabiensis*. *Anopheles arabiensis* is a relatively poor vector compared to *An. gambiae* s.s.[23], and its zoophilic and exophilic behavior moreover renders it less in contact with insecticide treated material (such as impregnated bed nets and insecticide treated walls) thus keeping it at a reduced risk of insecticidal pressure [37]. The increased role of *An. merus* as a potential vector also presents a challenge in the control as this particular mosquito is strongly exophilic, breeds in extensive brackish salt water, and is difficult to control with treated materials and larvicides. Moreover, since the *An. merus* population peaks during the dry period (when the salt concentration is optimal) while the population of fresh water species diminishes, this vectorial system will sustain transmission for a much longer period. While these considerations are valid for both malaria and lymphatic filariasis, the effect of the shift in *An. gambiae* s.l. sibling species composition on transmission of *W. bancrofti* is even more complex, as its transmission in East Africa involves both anophelines (*An. gambiae* s.l., *An. funestus*) and culicines (*Culex quinquefasciatus*). Although the anophelines in this area are more efficient lymphatic filariasis vectors than the culicines [7,19,45,46], an observed increasing number of culicines may to some extent compensate for the decreasing number of anophelines. It is therefore likely that the decline in anopheline mosquitoes will have comparatively less impact on the transmission of lymphatic filariasis than that of malaria.

## Conclusions

The findings confirm that the members of the *An. gambiae* s.l. complex reported in the past are still present in the study area although a clear shift in the sibling species composition has taken place. Combined with the earlier reported dramatic decline in overall anopheline vector density, the study suggests that this decline has been most marked for *An. gambiae s.s.*, and least for *An. arabiensis*, leading to current predominance of the latter. Due to differences in ecological requirements and vectorial capacity of the *An. gambiae* s.l*.* complex sibling species, the change in their composition undoubtedly has important implications for the epidemiology and strategies for control of both malaria and lymphatic filariasis in the study area. Further studies are urgently needed to monitor and assess the changes taking place in these vector populations, and to elucidate the underlying causes, in order to understand more clearly the consequences for transmission and control of these infections.

## Competing interests

The authors declare that they have no competing interests.

## Authors’ contributions

YAD, MA, KMH, DWM, SMM, EMP and PES conceived the study and participated in its design. YAD coordinated the field and laboratory work, and drafted the manuscript with contributions from PES, MA and DWM. All authors read and approved the final manuscript.
